# Optimization Design of the Inner Structure for a Bioinspired Heel Pad with Distinct Cushioning Property

**DOI:** 10.3390/bioengineering10010049

**Published:** 2022-12-30

**Authors:** Jianqiao Jin, Kunyang Wang, Lei Ren, Zhihui Qian, Xuewei Lu, Wei Liang, Xiaohan Xu, Shun Zhao, Di Zhao, Xu Wang, Luquan Ren

**Affiliations:** 1 Key Laboratory of Bionic Engineering, Ministry of Education, Jilin University, Changchun 130025, China; 2 Weihai Institute for Bionics, Jilin University, Weihai 264402, China; 3 School of Mechanical, Aerospace and Civil Engineering, University of Manchester, Manchester M13 9PL, UK

**Keywords:** bionic design, heel pad, cushioning effect, finite element analysis, human walking

## Abstract

In the existing research on prosthetic footplates, rehabilitation insoles, and robot feet, the cushioning parts are basically based on simple mechanisms and elastic pads. Most of them are unable to provide adequate impact resistance especially during contact with the ground. This paper developed a bioinspired heel pad by optimizing the inner structures inspired from human heel pad which has great cushioning performance. The distinct structures of the human heel pad were determined through magnetic resonance imaging (MRI) technology and related literatures. Five-layer pads with and without inner structures by using two materials (soft rubber and resin) were obtained, resulting in four bionic heel pads. Three finite element simulations (static, impact, and walking) were conducted to compare the cushioning effects in terms of deformations, ground reactions, and principal stress. The optimal pad with bionic structures and soft rubber material reduced 28.0% peak vertical ground reaction force (GRF) during walking compared with the unstructured resin pad. Human walking tests by a healthy subject wearing the 3D printed bionic pads also showed similar findings, with an almost 20% decrease in peak vertical GRF at normal speed. The soft rubber heel pad with bionic structures has the best cushioning performance, while the unstructured resin pad depicts the poorest. This study proves that with proper design of the inner structures and materials, the bionic pads will demonstrate distinct cushioning properties, which could be applied to the engineering fields, including lower limb prosthesis, robotics, and rehabilitations.

## 1. Introduction

The feet play a significant role in the normal gait of the human body, especially for shock absorption during contact with the ground [1,2]. The great impact resistance of the heel pad is an important specification which could be implemented in the fields of prosthetics, rehabilitation products, and robotics. For patients with amputations, the loss of foot can have a significant impact on daily life and work, so the design and development of prostheses are becoming increasingly important. Generally, there are three main methods used to absorb and dissipate the ground reactions in conventional prosthetics [3,4,5,6]. The first is the elastic element assembled on the bottom of the prosthetic footplates. Nickel et al. [3] designed a prosthesis that simply adds rubber to the bottom of the footplate to increase the cushioning effect. The second is the deformation of the carbon fiber plate itself. Song et al. [4] designed an arched footplate to increase cushioning through deformation. The third is the expansion and contraction deformation of the rigid spring. Collins et al. [5] designed a prosthesis with energy storage function, which can achieve good cushioning performance by compressing the spring.

Heel pain has become a common muscle and bone disease in chronic diseases. [7,8]. When patients walk for a long time or stand in an incorrect position, various foot trauma and inflammation can occur, such as plantar fasciitis, heel pad syndrome and calcaneal stress fracture, which seriously affects the quality of daily life [1,2,9]. Therefore, how to treat heel pain and reduce secondary injuries is becoming increasingly crucial. Diabetic foot is one of the severe complications of diabetes and is a series of secondary foot lesions caused by high blood sugar [10]. By studying the foot tissues of diabetic foot patients and healthy people of the same age, no significant difference in stiffness was found between the two, but the impact of the heel pad of diabetic foot patients was higher than that of healthy people [11]. It can be seen that wearing a suitable cushioning insole can effectively reduce the pressure on the foot soles and prevent further damage to the feet.

Most existing prosthetic products use simple flexible materials to replace the heel, but fail to consider the distinct cushioning effect (i.e., impact resistance) of the heel pad. The above three common methods of increasing cushioning performance are relatively simple and easy to implement in structure, but they have some intrinsic defects. Soft elastic elements and carbon fiber footplates will increase the energy stored and returned during the gait cycle. However, the pre-pressure will affect the natural movement of the ankle joint and easily increase the initial high loading rate, resulting in instability and inadaptability of the amputee. Although the energy consumption of the spring heel is relatively small, the excess energy will return to the human residual limb and cause secondary damages [6,12,13].

On the other hand, biped robots are currently widely used in various complex working environments due to high terrain adaptability and flexibility. However, there are still many problems in the field of robotics. One of the key points is how to reduce the impact faced by the robot when moving and improve the cushioning performance of the robotic foot [14,15]. In previous studies, Sun et al. [16,17] increased the compatibility of each mechanism during motion through the optimization and compensation of the control algorithm, thereby reducing the impact on the robot foot. Hamill et al. [18] designed a passive buffering structure composed of linear springs to enhance the cushioning of robot movement. Zhang et al. [19] proposed a two-degrees-of-freedom unit, which can reduce the impact through the flexible structure and the deformation of the compression spring. Although these studies can well reduce the impact force, the design of additional mechanisms will increase the energy consumption and weight of the robot, further adding extra costs of manufacturing and use.

As a unique structure, the heel pad is optimized mainly for bearing the weight of the human body. The heel pad of a healthy person is located on the underside of the rear bone (calcaneus) and is made up of fat cells and numerous fibrous tissue membranes [20,21,22,23,24]. The fibrous elastic tissue diaphragm of the heel pad is arranged in a honeycomb shape, which has strong mechanical strength. The compact and firm structural features play a role in separating fat cells and supporting protection, and it is not easy to produce excessive movement when subjected to the human body load. In the histological examination, the fibrous diaphragm in the central part is smaller than that in other parts. This structure is more convenient to increase the degree of strain in the highest stress area [25,26,27,28,29,30]. Compared with other fat structures in the human body, these characteristics of the heel pad endow it with better cushioning performance [31], allowing it to become an effective shock absorber for the human body [32]. The human heel pad can absorb the ground reactions when the heel touches the ground in the early normal gait and protect the bones against secondary damage caused by vibration [33,34].

Therefore, this study aims to develop a bioinspired heel pad with passive cushioning characteristics by extracting and simulating the inner structures of the human heel pad. Four types of bionic pads with/without five-layer inner structures were designed using two different materials. Three finite element simulations (static, impact, and walking) were carried out to optimize the design by comparing the cushioning effects in terms of deformations, ground reactions and principal stress. Finally, human walking experiments were performed by wearing the bionic pads with five-layer cushioning structures to validate the simulation results. In the future, the bionic pads can be applied to such fields as lower limb prosthesis, robotics, and cushioning insole design.

## 2. Materials and Methods

### 2.1. Medical Imaging of Human Heel Pad

**Figure 1:**
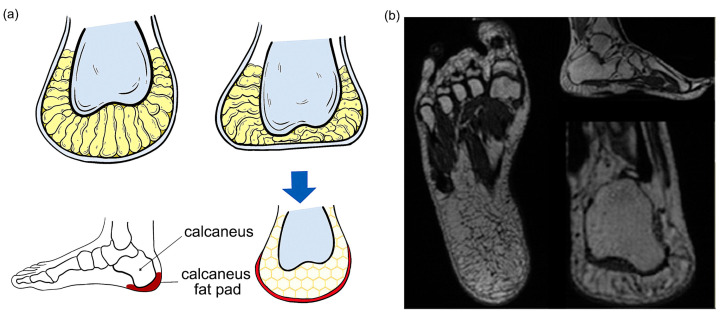
Human heel pad and its magnetic resonance imaging (MRI) images. (**a**) Indication of nonstress and stress on the heel pad of the normal foot. The position and the honeycomb structure of the heel pad were depicted; (**b**) MRI image of the human heel pad.

In this study, magnetic resonance imaging (MRI) technology was used to determine the inner structures of the human heel pad (Figure 1a). The MRI images of the heel pad (Figure 1b) were collected from a healthy man (age: 30 years, weight: 75 kg; height: 175 cm) with no history of musculoskeletal disease by using a GE Discovery 750 MRI system (General Electric, Boston, MA, USA). The thickness of the scanning layer is 0.5469 mm and the cross-sectional resolution is 512 pixels ×512 pixels. The scanned data were saved in Dicom format. The subject provided written informed consent before participating in the MRI scans and plantar stress tests. This study was approved by the Ethics Committee of the Second Hospital of Jilin University (Log# 2021072), in accordance with the Declaration of Helsinki (2013) and Biomedical Research Involving Human Subjects International Code of Ethics for Research (2002).

### 2.2. Structure Design of Bionic Heel Pads

The average thickness of a normal human heel pad is approximately 18 mm [20,25] (Figure 1b). From the top (near the root bone) to the bottom, the human heel pad is composed of five parts: deep subcutaneous layer, superficial subcutaneous layer, elastin-rich reticular dermis, collagen-rich dermis, and thick epidermis. Among these parts, the deep subcutaneous layer has a wide interval, the fibers of the subcutaneous tissue are only the components that form the “diaphragm”, and the diaphragm forms the “chamber”. There are some fibers that do not form a septum in the subcutaneous tissue and septa that do not restrict the chamber [35,36]. The deep subcutaneous layer and the superficial subcutaneous layer are connected by a collagen diaphragm. The inner diaphragm fiber structure of the superficial subcutaneous layer is smaller and thinner, including elastic tissue and a small amount of collagen fibers. Due to the difference in elastic modulus, the superficial subcutaneous layer is almost incompressible and mainly plays a bearing role [37], while the protein content of the deep subcutaneous layer is thicker than the protein content of the superficial subcutaneous layer (Figure 1a).

**Figure 2:**
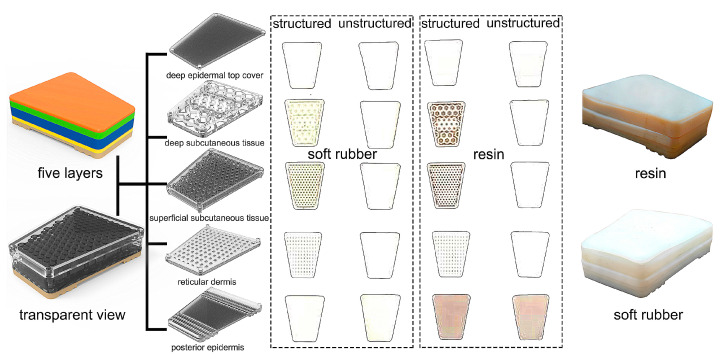
Design of the four bionic heel pads with different inner structures and materials.

Combining the literature with the structure, morphology, and characteristics of the heel pad obtained from the MRI images, the main elements and design regularization of the bionic pad were extracted and refined. The overall shape of the bionic pad is regularized into a trapezoid according to the shape of the human heel pad, and the inner structures have five layers (Figure 2), which are the deep epidermal top tissue (deepest), deep subcutaneous tissue, superficial subcutaneous tissue, elastin-rich reticular dermis tissue, and posterior epidermis (most superficial). The human heel pad contains a honeycomb structure, two mesh structures (one larger diaphragm shape and one smaller chamber shape), and other regular shapes. For the bionic pad, the honeycomb structure with non-uniform hexagonal units of different sizes was designed in the deep subcutaneous layer, two quadrilateral structures of different sizes were implemented in the network of the superficial subcutaneous tissue and elastin-rich reticular dermis tissue. The deepest and most superficial layer were unstructured with uniform material properties.

To compare the cushioning performance, two types of design, with and without internal structures, were finalized (Figure 2). It should be noted that the unstructured design refers to the absence of any internal structure, such as honeycomb barriers and mesh structures, except for the same material hardness. To enhance the superiority of the structural design, the thickness of the bionic heel pad was designed to be 20 mm (2 mm thicker than the human heel pad). The length of the heel pad was 79 mm, the short width and long width were 32 mm and 58 mm, respectively. It should be noted that the performance of the bionic pad was mainly determined by its thickness and inner structures. The dimensions in terms of short width, long width, and length were not the main performance factors, so they could be adjusted according to different user environments. 

### 2.3. Finite Element (FE) Simulation

To compare the mechanical properties of the four bionic heel pads in terms of different inner structures and materials, finite element simulations were conducted in ABAQUS (Dassault Systèmes, Waltham, MA, USA). The heel pads were meshed by 4-node tetrahedron (C3D4) elements. After mesh sensitivity analysis, the mesh size is set to 2 for the following analysis. The overall mesh quantity and material properties of different pads were listed in Table 1. Tie constraints were applied between the five layers of the pad. Three different simulations were conducted: static, impact, walking. In the static test, a concentrated force of 1500 N was uniformly applied on the top of the bionic pad, and the bottom of the pad was fixed. The principal stress and spatial deformation were obtained. In the impact test, two rigid plates meshed by 8-node hexahedral element (C3D8I) elements were put on the top and bottom of the pad, respectively. Tie constrains were applied between the plates and the pad surfaces. The lower plate was fixed, and the upper one was added with five different initial speeds (0.05 m/s, 0.1 m/s, 0.2 m/s, 0.3, and 0.4 m/s) for impact testing. The vertical displacement of the upper plate and the vertical ground reaction forces (GRFs) on the lower plate were measured. In the walking test, four key time instants (heel strike, loading response, foot flat, and heel off) during human walking were selected as the reference, and the angles formed by the human foot with the ground in three planes (sagittal, coronal and horizontal) as well as the velocity and acceleration of the foot at each instant were input as the kinematic parameters. Tie constraints were applied between the pad and the ground. The principal stress and vertical GRFs of the four pads were then measured.

### 2.4. Material Selection and Prototype Manufacturing

As the available materials in market cannot perfectly match the characteristics of human heel pad, we use the proportional method to determine the different materials, that is to use the ratio of different structural harnesses to select the corresponding proportion of imitated materials of the designed bionic pad [38]. According to the literature [39,40], the elastic modulus of the superficial subcutaneous tissue and deep subcutaneous tissue of the heel pad of a normal person is approximately (61 ± 11) kPa and (26 ± 6) kPa, respectively. This ratio is approximately 2:1, which is also used in the selection of the structural harnesses of the different layers in the bionic pads. The Shore A hardness is used in this study as guideline because the commercial soft materials normally ranges from 10° to 90° in Shore A hardness. The hardness of the superficial subcutaneous tissue and the deep subcutaneous tissue materials of the bionic heel pad were selected to be 60 HA and 30 HA (2:1), respectively. The selection of other soft materials is detailed in the list of specific performance parameters (see Table 2). The thickness of each layer is based on the literatures and the IMR images observed in this study. 

Finally, five layers in terms of different structures were separately 3D printed by a PolyJet J850 system (Stratasys, Eden Prairie, MN, USA) with two different types of materials (resin and soft rubber) as shown in Figure 2. Small silicone round beads used to simulate human fat particles were added to the inside of the bionic pad. The five different layers were then assembled with each other through positioning posts and empty slots. To provide integrity of the bionic pad, DP100PLUS (3M, Two Harbors, MN, USA) soft glue was applied along the whole plane across the interface for bonding.

### 2.5. Human Walking Tests

Walking tests by the subject wearing customized shoes containing the designed bionic pads were conducted to investigate the influence of the structure and organization of the heel pad on the cushioning performance. The FootScan Plate (RSscan, Beringen, Belgium) was used to measure the ground reactions during walking gait. According to the size of the heel pad structure designed in this study, the pad is fixed on the root of the shoe to ensure smoothness when the foot touches the plate. The subject was briefly updated with the corresponding training to ensure the smooth progress of the experiment and the collection of relatively stable data. Before testing, static calibration and debugging of the plate were performed. The subject was asked to walk on the plate in three self-selected speeds: slow (1.2 ± 0.2) m/s, normal (1.4 ± 0.3) m/s, and fast (1.8 ± 0.3) m/s. The subject does not directly walk up or down the plate but leaves a distance of one meter on the front and back sides of the plate, allowing to walk with a more natural gait when the instruction is started. Finally, each test was repeated 5 valid times under the same conditions for further analysis and comparison. 

### 2.6. Statistical Analysis

The results of human walking experiments are shown as mean ± standard deviation. This research uses IBM SPSS Statistics 25 software (IBM, Armonk, NY, USA) for data significance analysis. All the *p* values of the univariate analysis are less than 0.001, indicating that both the structure and the material have statistically significant effects on the ground reaction forces. All data are presented at a *p* < 0.05 significance level unless otherwise stated.

## 3. Results

### 3.1. FE Static Analysis

**Figure 3:**
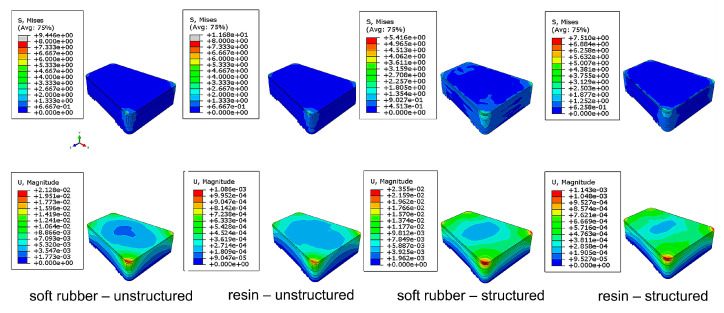
Distributions of the maximum principal stress (**top**) and the spatial displacement (**bottom**) of the four bionic heel pads under a 1500-N concentrated load in FE simulations.

Figure 3 and Table 3 show the stress and deformation of the four bionic pads under 1500-N compression load. The larger the deformation value is and the smaller the principal stress is, the better the effects of the shock absorption and buffering are. The results showed that the stress of the structured soft rubber sample was the smallest (5.41 MPa), and its deformation was the largest (2.35 × 10^−2^ mm). There is no significant difference between the stress of the unstructured soft rubber and the unstructured resin samples, but the deformation value of the former is more conspicuous (increased one order of magnitude than the latter). Through comparison between the structured and unstructured resin samples, the stress of the structured one was 35.7% smaller than the unstructured one, while the deformation was 945.9% larger. This indicates that the inner structures can greatly increase the cushioning performance of the bionic heel pad.

### 3.2. FE Impact Analysis with Different Speeds

**Figure 4:**
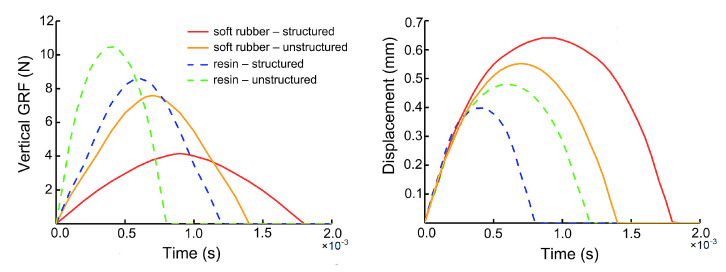
Vertical GRF and displacement (upper plate) of the four bionic heel pads under impact with an initial speed of 0.4 m/s in FE simulations.

The mechanical properties of the bionic heel pads under external impact was systematically studied by finite element analysis. Firstly, the vertical GRF and the deformation (expressed as the vertical displacement of the upper plate on the heel pad model) for each bionic pad (Figure 4) at the same velocity (0.4 m/s) were analyzed. When the same soft rubber material was used, the vertical GRF of the bionic pad was 43% lower than the that of the unstructured one, and the upper plate displacement was 18% higher. In addition, when the same resin material was used, the heel pad with the bionic inner structures had 19% lower vertical GRF and 15% greater upper plate displacement than that without the inner structures. The heel pad with the bionic structures was found to have superior cushioning performance due to its smaller vertical GRF, longer reset time, and higher deformation, while the cushioning effect of the soft rubber was higher than the that of the resin material.

**Figure 5:**
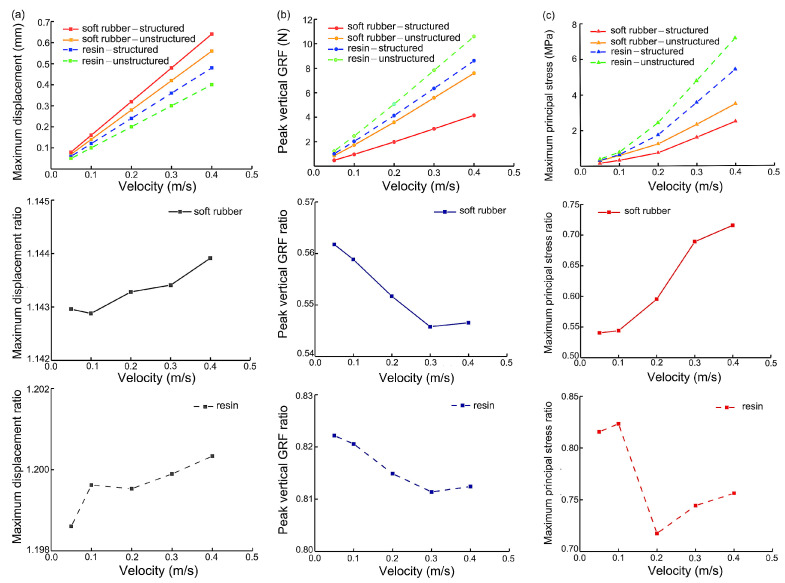
Maximum vertical displacement (**a**), peak vertical GRF (**b**), and maximum principal stress (**c**) of the four bionic heel pads under impact of different speeds in FE simulations. Maximum values (**top**) with different structures and materials, along with the ratio of the maximum value of the structured to the unstructured sample using same soft rubber (**middle**) or resin (**bottom**) material were depicted.

Then, we investigated the cushioning performance of the four heel pad structures at different impact velocities of 0.05 m/s, 0.1 m/s, 0.2 m/s, 0.3, and 0.4 m/s (Figure 5). The simulation results showed that for all the four heel pads, the maximum displacement of the structured was greater than that of the unstructured (Figure 5a), the peak vertical GRF was lower (Figure 5b), and the maximum principal stress (Figure 5c) was smaller. When the impact speed changes, the rates of variation of the above variables behave different (approximately linear for the displacement and GRF, nonlinear for the principal stress). For instance, the slope of the linear line connecting the peaks GRF of the unstructured was greater than that of the structured by using both soft rubber and resin materials (Figure 5b). More specifically, the slope of the soft rubber heel pad with the bionic structures was 59% lower than that of the unstructured, while the slope of the resin heel pad with the bionic structures was 21% lower than that of the unstructured. With increasing velocity, the value of the maximum principal stress of the heel pad with the bionic structures was smaller than that without the bionic structures (Figure 5c). However, the ratio changes of the stress of the structured to the unstructured samples under various impact speeds depicted different trends in the two materials. For soft rubber heel pads, the ratio was monotonically increasing with velocities, but for resin materials, the ratio had a dramatic decrease at the speed of 0.2 m/s. Further analysis showed that for both bionic pads with and without the bionic structures, the cushioning performance of the soft rubber was still found to be superior to that of the resin material. Generally, the soft rubber pads demonstrated higher displacement as well as lower vertical GRF and principal stress. The ratios of the maximum value of the structured to the unstructured in terms of these three parameters for soft rubber materials were better than those of resin materials, such as 0.56 (soft rubber) versus 0.82 (resin) in peak GRF at speed of 0.1 m/s.

### 3.3. FE Walking Gait Analysis

**Figure 6:**
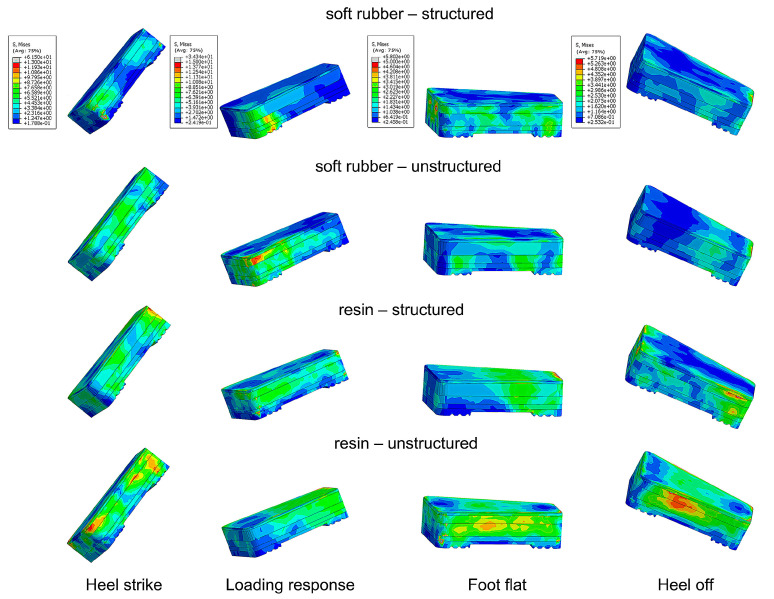
Distributions of the maximum principal stress of the bionic heel pads at four key time instants during normal walking gait in FE simulations.

**Figure 7:**
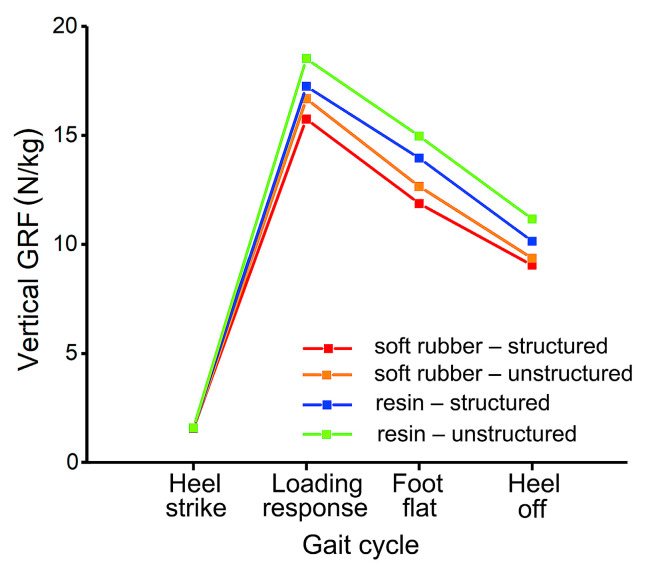
Vertical GRF of the four bionic heel pads at four key time instants during normal walking in FE simulations.

The stress and GRF of each bionic heel pad at four key events (heel strike, loading response, foot flat, and heel off) during normal walking were analyzed (Figure 6 and Figure 7). The maximum stress of all the heel pads at heel strike and loading response were much higher (almost ten times) than the values at foot flat and heel off. The lines connecting the vertical GRFs at the four time instants showed a type of convex shape, and at each instant, the values of the pads with the bionic structures were smaller than that without the bionic structures (Figure 7). The vertical GRF of the pad made of soft rubber was smaller than that of the resin pad, indicating that the cushioning performance of soft rubber was superior to that of the resin material. The largest vertical GRF occurred at the instant of loading response, while the soft rubber-structured, soft rubber-unstructured, resin-structured, and resin-unstructured pad had the values of 12.41 N/kg, 13.36 N/kg, 15.11 N/kg, 17.23 N/kg, respectively. The largest vertical GRF for the resin without the bionic structures increased by 38.9% compared to the minimum value of the soft rubber with the bionic structures. It can be concluded that among the four bionic pads, the structured soft rubber one has the best cushioning effect during human normal walking.

### 3.4. Physical Tests of Human Walking

**Figure 8:**
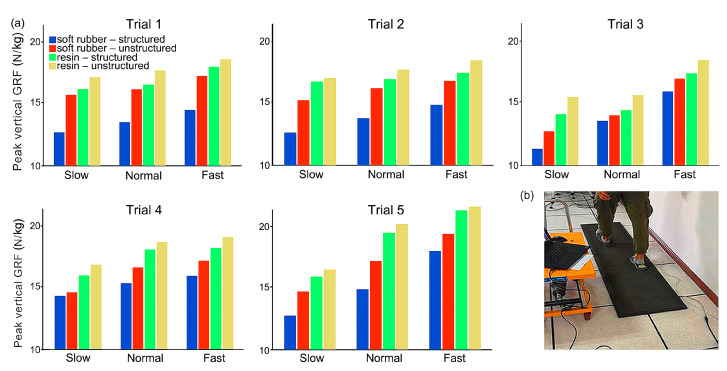
(**a**) Vertical GRF of all the five trials (*n* = 5) during human walking at different speeds; (**b**) Setups for human walking tests wearing the bionic pads.

**Figure 9:**
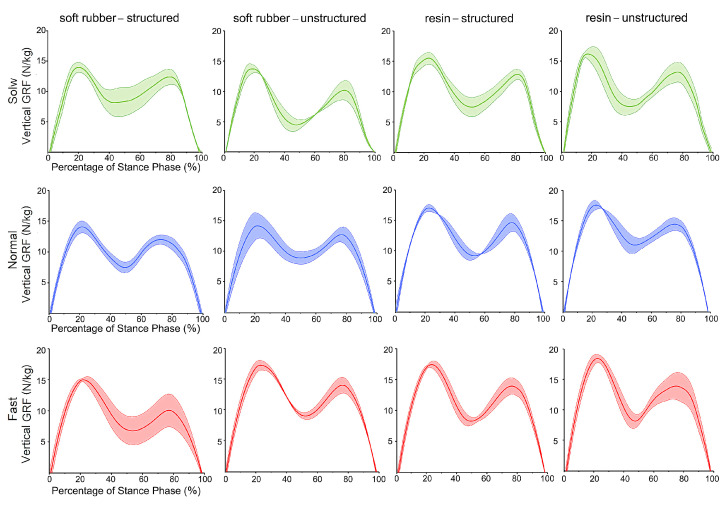
Time trajectories of the vertical GRF of the four bionic heel pads in one walking gait cycle at different speeds. Mean ± standard deviations were depicted, *n* = 5.

Figure 8 shows the peak vertical GRF of all the five trials during human walking at three different speeds for the four bionic pads, while Figure 9 depicts the time trajectories of the vertical GRF at each speed. Generally, the bionic pad made of soft rubber was better than that of resin, i.e., smaller peak vertical GRF in the soft rubber pad when the inner structure and speed were constant. In the case of the same speed and material, the heel pad with the bionic structures has better cushioning performance than the unstructured. The peak vertical GRFs configured with structured soft rubber pad were 13.13 N/kg, 13.88 N/kg, and 14.77 N/kg at slow, normal, and fast speeds, respectively, which were lower than those of the unstructured with 15.89 N/kg, 16.31 N/kg, and 17.28 N/kg, respectively. The decreases in the peak vertical GRFs at three speeds were 17.4%, 14.9%, and 14.5%, respectively. In addition, the walking speed has a certain impact on the cushioning effect. The slower the speed is, the lower the peak vertical GRF is, representing a better cushioning performance. In the same gait cycle, the vertical GRF gradually increased with the increase of speed. 

**Figure 10:**
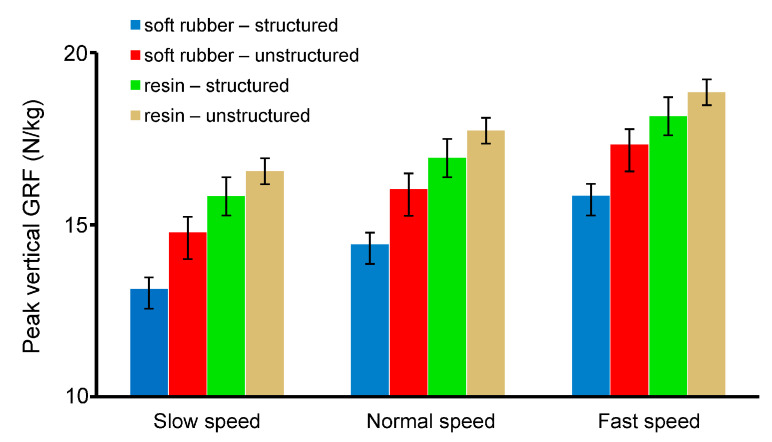
Mean and deviations of the peak vertical GRF of the four bionic heel pads during human walking at different speeds, *n* = 5.

Figure 10 shows the mean and standard deviations of the peak vertical GRF during human walking at three speeds configured with the four bionic heel pads. The largest is in the unstructured resin heel pad, and the smallest occurs in the structured soft rubber pad for all the three speeds. The peak vertical GRF in the structured soft rubber sample was 20.7%, 18.7%, and 16.8% smaller than that in the unstructured resin sample at slow, normal, and fast speed, respectively. This indicates that the cushioning effect of the heel pad with the bionic structures is better than that of the one without the inner structures, and the effect of the soft rubber material is greater. These findings are consistent with the FE simulation results of human walking. In conclusion, by mimicking the inner structures of the human heel pad, the bioinspired heel pad with five-layer structures is indeed helpful to enhance the cushioning effect during contact with the ground.

## 4. Discussion

In the normal anatomical structure of the human body, the heel pad has good cushioning performance due to its unique inner structures. However, in the design of the existing prosthetic footplates, rehabilitation insoles, and robotic feet, the cushioning performance is not well considered, and the deformation of a single elastic material and the movement of a simple mechanism are basically used to achieve the impact resistance. Specifically, the ground reaction force is the reaction force experienced by the human or robotic foot when the foot touches the ground. Excessive peak vertical GRF during walking will have a greater impact on the human body which can cause secondary damage to the musculoskeletal system. Therefore, the peak GRF is selected as a key index in this study to evaluate the quality of cushioning performance. 

Four bioinspired heel pads with certain inner structures were designed in this study through the morphological analysis of the human heel pad. The inner structure consists of five parts corresponding to the five tissue layers of the human counterparts. The cushioning performance of the designed pads was explored by means of three finite element simulations and a physical test of human walking. In addition, in order to further understand the impact of different materials on the cushioning performance, two materials of resin and silicone were selected for comparative analysis. It was found that the soft rubber heel pad with the bionic structures had the best cushioning performance (smallest peak vertical GRF and stress, largest deformation), followed by structured soft rubber and structured resin samples, while the resin heel pad without inner structures showed the poorest cushioning effect. Comparing the optimal pad containing both the bionic structures and soft rubber material to the unstructured resin pad, the peak vertical GRF during normal walking was reduced by 28.0% in simulations and 18.7% in the physical test. The results show that the bionic structures proposed in this study can effectively increase the cushioning performance, and the selection of the appropriate material will also enhance the cushioning effect.

Other researchers have previously designed different types of bionic pads based on different biological prototypes. Through the simulation of ostrich feet, Han et al. [38] developed bionic pads with different elastic modulus and compared their performance. Based on the foot of the German Shepherd Dog, Miao et al. [41] designed the bionic pad and discussed its cushioning performance by finite element analysis and physical testing. By comparing our findings with their results, we can see that the decrease of GRF during walking is improved by combining the inner structures and proper materials in this study (see Table 4).

Future works are prepared to further optimize the experimental process and continue to improve the accuracy and standardization of data. For instance, more accurate gait measurement should be conducted by using force plates which could observe the six degrees of freedom ground reaction forces and moments. Moreover, more physical experiments will be conducted to validate the simulation results, and the cyclic performance of each type of the four heel pads will be evaluated. More subjects with different body weight and foot size will be included to expand the applied range. In addition, more reliable options will be chosen by comparing other soft materials while processing the remaining Shore hardness materials of the same proportion to enrich the theoretical basis of this design.

## 5. Conclusions

This study developed bionic heel pads inspired by the optimized inner structures of the human heel pad to be used in the future design of prosthetic footplates and rehabilitation insoles and to provide feasible ideas for the cushioning parts of robotic feet. We first extracted the key structure, morphology, and characteristics of the five-layer human heel pad combing the MRI images and the literature. Then, the main elements and design regularization of the five-layer pad were established and optimized by changing the inner structures and materials, resulting in four types of bionic heel pads. Finally, we conducted three finite element simulations and physical human walking tests using a healthy subject. Simulations results showed that the heel pad with the bionic structures has superior cushioning effect with higher deformation, as well as smaller vertical GRF and principal stress. The cushioning performance of the soft rubber is higher than that of the resin material. Specifically, the peak vertical GRF during normal walking could be reduced by up to approximately 30% by the proper design of the inner structures and materials. The physical tests of human walking also depicted similar findings, with an almost 20% decrease in peak vertical GRF at normal speed. This study demonstrates that the bionic heel pad designed by combining the internal structures with a proper selection of materials can achieve expected cushioning performance. 

## Figures and Tables

**Table 1:** Mesh quantity and material properties of the four bionic heel pads.

Name	Soft Rubber-Structured	Resin-Structured	Soft Rubber-Unstructured	Resin-Unstructured
Mesh quantity	92,653	92,653	32,048	32,048
Young’s modulus (MPa)	6	12	6	12
Poisson’s ratio	0.47	0.3	0.47	0.3

**Table 2:** The thickness and hardness of the five layers of the bionic heel pad.

Position	Name	Thickness (mm)	Shore Hardness (HA)
Top layer (Deepest)	Deep epidermal top cover	2	30
Second layer	Deep subcutaneous tissue	5	30
Third layer	Superficial subcutaneous tissue	6	60
Fourth layer	Reticular dermis	3	50
Bottom layer (Most superficial)	Posterior epidermis	4	80

**Table 3:** Maximum principal stress and deformations of the four bionic pads under a 1500-N concentrated load in FE simulations.

Name	Soft Rubber-Structured	Resin-Structured	Soft Rubber-Unstructured	Resin-Unstructured
Max. stress (MPa)	5.41	7.51	9.45	11.68
Max. deformation (mm)	2.35 × 10^−2^	1.14 × 10^−2^	2.12 × 10^−2^	1.09 × 10^−3^

**Table 4:** Comparison of different bionic pads.

Category	Han et al. [38]	Miao et al. [41]	This Study
Bionic prototype	Ostrich	German Shepherd Dog	Human
Internal structures	No	Yes	Yes
Multiple materials	Yes	No	Yes
Cushioning performance	Impact force decreased by 17%	GRF decreased by 14%	GRF decreased by 20%

## Data Availability

The datasets generated during and/or analyzed during the current study are available from the corresponding author on reasonable request.
